# On the knowledge of solitary juvenile xanthogranuloma of the eyelid: a case series and literature review

**DOI:** 10.1007/s00417-022-05560-6

**Published:** 2022-01-27

**Authors:** Rongxin Chen, Shu Liu, Lijuan Tang, Xinyue Yu, Ziwei Meng, Yu Hu, Jing Li, Xuanwei Liang

**Affiliations:** 1 State Key Laboratory of Ophthalmology, Zhongshan Ophthalmic Center, Sun Yat-sen University, Guangdong Provincial Key Laboratory of Ophthalmology and Visual Science, Guangdong Provincial Clinical Research Center for Ocular Diseases, Guangzhou, 510060 China; 2 Department of Pharmacy, State Key Laboratory of Oncology in South China, Collaborative Innovation Center for Cancer Medicine, Sun Yat-sen University Cancer Center, Guangzhou, 510060 China

**Keywords:** Solitary, Eyelid lesion, Juvenile xanthogranuloma, Age at onset, Treatment

## Abstract

**Purpose:**

Solitary eyelid juvenile xanthogranuloma (JXG) is extremely rare, and there is limited literature on its clinical features and treatment outcomes. Here, we present a case series and comprehensive review of the literature on patients with isolated eyelid JXG.

**Methods:**

We systematically extracted data from our institution’s records of isolated eyelid JXG cases and conducted a search for additional cases from the literature utilising the PubMed, Wanfang, and Chinese National Knowledge Infrastructure (CNKI) databases. Patients with JXG were analysed with respect to age, sex, clinical presentation, therapy, and outcome. Group comparisons were performed.

**Results:**

Thirty-two patients (including 13 at our institution and 19 from prior publications) were identified. The median age at first presentation was higher in current patients than in the patients from the published cases (median 9 years, range 1.2 to 47.0 years; median 2 years, range 0.5 months to 46.0 years, respectively, *P* = 0.014). Of the patients who had known characteristics, no significant differences were observed between the two groups in terms of sex, affected eye, eyelid site, type of cutaneous involvement, or duration of symptoms (each *P* > 0.05). Seventeen (54.8%) patients were male. The most common lesion location was the upper eyelid (*n* = 10, 62.5%). Twenty-four (75.0%) cutaneous lesions had full-thickness skin involvement; 8 (25.0%) subcutaneous masses had a chalazion-like appearance. Histologically, the JXG masses were characterised by Touton giant cells with inflammatory cells. Additionally, there was no significant difference in treatment modalities between the two groups (*P* = 0.072), and 24 (75.0%) patients underwent surgical excision. The overall recurrence-free survival was 3.6 to 52.8 (median 27.0) months in the current patients. For published cases with available follow-up information, there was no recurrence in 10 cases and improvement in 1 case, with a median follow-up of 9.5 months.

**Conclusion:**

Solitary eyelid JXG is a rare clinical entity and should be included in the differential diagnosis of eyelid mass lesions in patients of all age groups. Surgical excision is often selected for efficient treatment and to obtain an excisional biopsy.

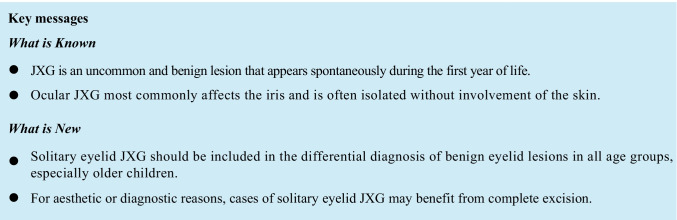

**Supplementary Information:**

The online version contains supplementary material available at 10.1007/s00417-022-05560-6.

## Background

Juvenile xanthogranuloma (JXG) is the most common form of non-Langerhans cell histiocytosis, and it usually presents in infancy and early childhood. Up to 90% of the JXG cases are cutaneous lesions occurring in the head and neck, which are the most common sites that are involved [1–4]. Although adults may also be affected [5], the lesions mostly occur spontaneously within the first year of life as cutaneous nodules that can be yellow or a range of other colours [6], and they can regress spontaneously [7]. JXG has been reported to represent 0.5% of all paediatric tumours [8]. The patient’s general health is not impaired, and in the absence of associated conditions, the prognosis is excellent. Extracutaneous JXG most commonly affects the eye but can occur in the bone, spleen, brain, lung, liver, and other sites [9–13]. Ocular involvement with JXG is rare and is estimated to occur in approximately 0.3 to 10% of patients with cutaneous JXG [7, 14]. In particular, extremely rare cases of solitary eyelid lesions have been reported.

In this study, we identified patients at our institution with solitary JXG of the eyelid and evaluated their clinical details with respect to age, clinical presentation, involved sites, therapy, and outcome. Moreover, a comprehensive review of all prior English or Chinese language publications on solitary JXG of the eyelid and its comparisons with our case series has been performed here.

## Methods

### Institutional case series

This study was approved by the Institutional Review Board at Zhongshan Ophthalmic Center, Sun Yat-sen University, Guangzhou, China. We searched the surgical pathology system from June 2016 to July 2020 for patients with biopsy-proven JXG. Immunohistochemistry was performed using the following antibodies: CD 68, CD1, and S-100. Inclusion criteria included patients who were diagnosed with isolated JXG of the eyelid. Exclusion criteria included those patients with any additional ocular or systemic lesions. During the period between June 2016 and July 2020, 17 patients with JXG were sampled at our institution; however, we identified 13 patients diagnosed with solitary JXG of the eyelid.

### Literature review

We conducted a literature search utilising PubMed, Wanfang, and the Chinese National Knowledge Infrastructure (CNKI). The search strategy used the following terms: eyelid JXG, eye JXG, and skin JXG. We included publications in the English or Chinese language, and we included patients with solitary JXG of the eyelid, regardless of the patient’s sex, age, affected eye, eyelid site, type of cutaneous involvement, symptoms, duration of symptoms, treatment modalities, and prognosis. We excluded any patients with lesions occurring at other ocular sites, and we excluded any patients with a known history of JXG that appeared elsewhere on the body. Relevant articles were used for comparison with the current cases.

### Statistical analysis

The median (range) or frequency (percentage) was calculated for each clinical characteristic factor. As nonparametric significance tests, the Mann–Whitney *U* test, chi-square test, or Fisher’s exact test was performed when appropriate. Statistical analyses were performed using IBM SPSS software (Version 20.0; SPSS Inc., Chicago, IL, USA), version 6. *P* < 0.05 was considered statistically significant.

## Results

### Clinical characteristics

Our institutional clinic records contained 13 cases of primary solitary JXG of the eyelid. The patients were not on any topical treatments or systemic medications. Laboratory investigations, including complete blood counts, liver function tests, and renal function tests, were negative in all of the patients, and no systemic anomalies were found. Other than the solitary JXG found in the patients’ eyes, the ophthalmic examinations were normal in all of the patients. In addition to the aforementioned case series, we identified 19 additional patients with solitary eyelid JXG in 18 prior publications (Table S1) for a total of 32 patients. In all of the patients, the lesions were limited to one eye only, and the contralateral eyes were normal in all of the patients. All patients had a complaint of an eyelid mass, and no similar cutaneous lesions were present elsewhere on the head, neck, trunk, or extremities.

**Fig. 1:**
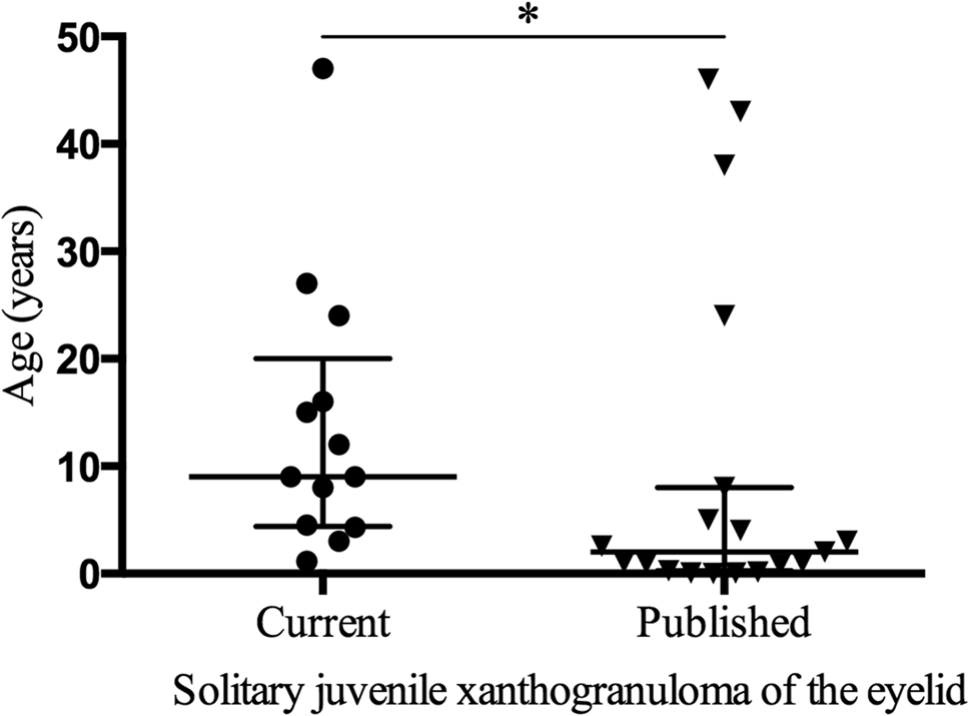
Age distribution between the current patients and published patients

**Fig. 2:**
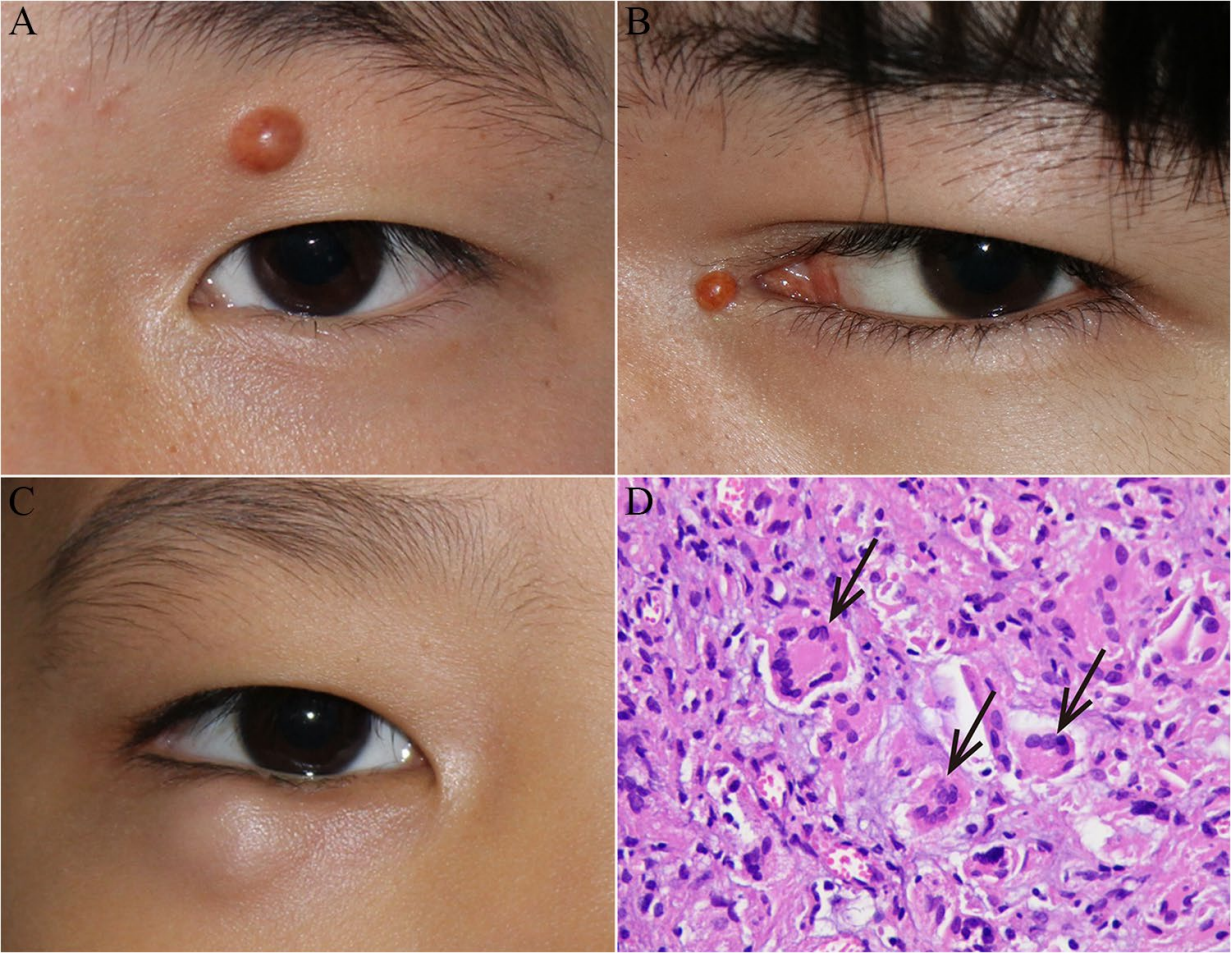
Clinical presentation and histopathology of isolated JXG of the eyelid. External photograph of a yellowish, solitary, and painless nodule located at the upper eyelid (**a**) or medial canthus (**b**) of the left eye. A 9-year-old boy had a right subcutaneous lesion with a chalazion-like appearance of the lower eyelid (**c**). Histopathological assessment revealed diffuse inflammatory cells, mostly histiocytes with scattered giant cells displaying Touton-type features (**d**) (arrow)

As shown in Fig. 1, the age (median, 9 years; range, 1.2 to 47.0 years) at first presentation was higher in current patients than in published patients (median, 2 years; range, 0.5 months to 46.0 years) (*P* = 0.014). Furthermore, of the patients who had known characteristics, no significant differences were observed between the two groups in terms of sex, affected eye, eyelid site, type of cutaneous involvements, or duration of symptoms (each *P* > 0.05, Table 1). A total of 54.8% (17/31) of patients were male, and 59.4% (19/32) of patients were affected in the right eye. The locations of the lesions included the upper eyelid (62.5%, *n* = 20), lower eyelid (31.25%, *n* = 10), and medial canthus (6.25%, *n* = 2). A total of 75.0% (24/32) of patients had cutaneous lesions with full-thickness skin involvement (Fig. 2a and b), while 25.0% (8/32) of patients had subcutaneous masses, showing a chalazion-like lesion on the eyelid (Fig. 2c). Of the 27 patients with available information, the duration of symptoms associated with the eyelid masses ranged from 0.5 to 12.0 months (median 6.0 months). In 2 of the published patients, the masses were chronic with multiple recurrences and had been present for at least 6 to 7 years.

**Table 1:** Characteristics of the patients with solitary JXG of the eyelid between the current cases and published cases

Clinical features	Patients of the current cases	Patients of the published cases	*P*
Age at first presentation (years)			
Median	9.0 years	2.0 years	0.014
Range	1.20 to 47.0 years	0.5 months to 46.0 years	
Sex, *n* (%)			
Male	8 (61.5)	9 (50.0)	0.524
Female	5 (38.5)	9 (50.0)	
Affected eye, *n* (%)			
Right	9 (69.2)	10 (52.6)	0.348
Left	4 (30.8)	9 (47.4)	
Eyelid site, *n* (%)			
Upper eyelid	7 (53.8)	13 (68.4)	0.732
Lower eyelid	5 (38.5)	5 (26.3)	
Medial canthus	1 (7.7)	1 (5.3)	
Type of cutaneous involved, n (%)			
Cutaneous	11 (84.6)	13 (68.4)	0.420
Subcutaneous	2 (15.4)	6 (31.60	
Mean duration of symptoms (months)			
Median	6.0	5.0	0.943
Range	0.5 to 8.0	0.6 to 12.0

In all of the patients, histopathologic studies (incisional biopsy or excisional biopsy) revealed a dense lymphohistiocytic infiltrate with scattered Touton giant cells (Fig. 2d). The specimens were negative for malignancy, negative or slightly positive for CD1 and S-100, and positive for CD 68. According to these results, all the patients were diagnosed with JXG.

### Treatment and outcome

The treatment and prognosis data of the patients are summarised in Table 2. The treatment modalities included surgical excision, incisional biopsy and corticosteroids (topical injection, periocular, and/or oral), laser ablation, radiotherapy, and observation. There was no significant difference in the treatment modalities between the two groups (*P* = 0.072), and 24 (75.0%) patients underwent surgical excision.

**Table 2:** The treatment modalities and prognosis of current and published cases

	Current cases	Published cases	*P*
Treatment modalities, *n* (%)			
Excisional biopsy	11 (84.6)	13 (68.4)	0.072
Incisional biopsy and steroids	0 (0)	4 (21.1)	
Laser ablation	2 (15.4)	0 (0)	
Radiotherapy	0 (0)	1 (3.1)	
Observation	0 (0)	1 (3.1)	
Outcomes, *n* (%)			
No recurrence	13 (100)	10 (90.9)	0.458
Regression in size	0 (0)	1 (9.1)	
Not available	0 (/)	8 (/)	

Of the current 13 patients, the median overall recurrence-free survival was 27.0 (range, 3.6 to 52.8) months. For the published patients with known follow-up information, there were no recurrences in 10 patients, there was improvement in 1 patient, and there was a median follow-up of 9.5 months. Of these patients, two patients had a history of multiple recurrences after excision and prior to publication, and these two patients were successfully treated by surgical excision combined with grafting and low-dose radiotherapy. However, 8 (42.1%) patients had unknown outcomes, or their prognosis was not reported when the cases were published.

## Discussion

JXG, a disease of unknown aetiology and pathogenesis, has attracted the attention of ophthalmologists for over half a century [15]. JXG is a rare and a typically benign lesion that often presents in children below five years of age, with 85% of the patients being under the age of 1 year [15]. The iris is the most frequent anatomical part of the eye that is affected by JXG [12]. To date, eyelid JXG is an uncommon presentation, only with several individual case reports in the literature [12, 16–32].

We described a case series of solitary eyelid JXG without any other cutaneous or systemic involvement. Although ocular JXG may occur without skin involvement, with 92% of the cases of JXG occurring in patients during the first 2 years of life [7], our study showed that more than half of the patients were older than 2 years of age at the diagnosis of a solitary eyelid JXG involving cutaneous JXG, and this was noted in both the current cases and the published cases. Eyelid lesions of JXG tend to present later than uveal lesions, but the development JXG in the third decade of life or older is rare.

Ocular JXG patients with cutaneous involvement always have multiple skin lesions. In contrast, the incidence of ocular involvement in cutaneous JXG patients was estimated to be 0.3 to 10% [7, 14]. Consistent with our report, there is a predilection for the involvement of both the skin and the eye, and lesions in the eyelid skin appear quite suddenly. The lesions are usually solitary lesions and are typically the only apparent manifestation of JXG. As in previous studies [5, 12], the eyelid is the most frequently anatomical area affected, followed by the iris and the conjunctiva.

Eyelid lesions in children are uncommon. In a survey of 398 excised eyelid lesions of children conducted by Doxanas et al. [33], the most common lesion was chalazion (20%), followed by dermoid cyst (16%), papilloma (14%), pyogenic granuloma (9%), melanocytic nevus (9%), haemangioma (7%), neurofibroma (2.5%), and molluscum contagiosum (2.5%). According to the report by Al-Faky [24], the most common benign eyelid lesion was sweat gland hidrocystoma followed by chalazion in patients aged 2 to 87 years. Based on the characteristic clinical features, we may be unaware of a subcutaneous JXG of the eyelid due to the presentation of a chalazion-like lesion in our study. JXG is the most commonly encountered cutaneous non-Langerhans cell histiocytosis [6]; however, Alkatan et al. [5] reported that only 33.7% of clinical diagnoses were in accordance with the histopathologic diagnosis. Therefore, solitary subcutaneous JXG involving the eyelid should be included in the clinical differential of eyelid lesions, and its diagnosis should be confirmed by histopathology, which is characterised by the presence of Touton giant cells in most cases of JXG.

Cutaneous JXG is a benign disorder in which a well-circumscribed dermal or dermohypodermal nodule sparing the epidermis is presented, and childhood JXG tends to be benign and self-limiting, usually regressing spontaneously over one or two years. The conservative management of these lesions has been advocated [34]. However, adult-type lesions are usually solitary and rarely resolve spontaneously [6]. Lesions that develop in patients who are 20 years of age or older (as in the previous case) may persist indefinitely [35]. According to the newly revised histiocytosis classification [6], the histopathology and phenotype of disseminated JXG are not different from those of Erdheim-Chester disease (ECD); however, JXG and ECD are non-Langerhans cell disorders arising from either a dendritic or a macrophage cell. Moreover, 20% of patients with ECD also have lesions of Langerhans cells histiocytosis [6]. ECD is an adult histiocytosis characterised by symmetrical long bone involvement, cardiovascular infiltration, retroperitoneal fibrosis, and central nervous system involvement. ECD is marked by a heterogenous clinical course with some patients having progressive and lethal disease [6]. Therefore, excision of the eyelid lesion is often chosen due to aesthetics or especially for diagnostic purposes, as was done in our report. According to a previous report that lesion excision is an adequate treatment [36], our patients with isolated eyelid JXG had no local recurrence following surgical excision with a median follow-up duration of 27.0 months. As previously reported, if recurrence occurs after a period of observation, surgical excision and a biopsy of each edge or low-dose radiotherapy may be effective to treat recurrent lesions [16, 20].

## Conclusion

Solitary eyelid JXG should be included in the differential diagnosis of benign eyelid masses in all age groups. The diagnosis of solitary eyelid JXG should be based not only on the clinical characteristics but also on histopathological features. Complete excision may benefit patients with solitary eyelid JXG.

## Supplementary Information

Below is the link to the electronic supplementary material.

Supplementary file1 (DOCX 37 KB)

## Data Availability

The data used for the analysis are available from the corresponding author upon reasonable request.
