# Supplementary material for: On the knowledge of solitary juvenile xanthogranuloma of the eyelid: a case series and literature review
Source: Graefes Arch Clin Exp Ophthalmol. 2022 Jan 27;260(7):2339–45. doi: 10.1007/s00417-022-05560-6 (PMC9203400; doi:10.1007/s00417-022-05560-6)
Supplement: Supplementary file 1 — Supplementary file1 (DOCX 37 KB) [file 417_2022_5560_MOESM1_ESM.docx]

**Supplemental Information**

**Table S1.** Summary of published case reports of solitary JXG of the eyelid.

| **No.** | **Author** | **Published date** | **Age (years)** | **Gender (1=female, 2=male)** | **Affected eye (1=right, 2=left)** | **Eyelid site (1=upper, 2=lower, 3=medial)** | **Cutaneous site (1=subcutaneous, 2=cutaneou)** | **Symptoms** | **Duration of symptoms (months)** | **Systemic manifestation (0=Not documented, 1=No systemic involvement)** | **Treatment (1=Excisional biopsy, 2=Incisional biopsy and steroids, 3=Laser ablation, 4=radiotherapy, 5=Observation)** | **Outcome (1=no recurrence, 2=decreased in size, 3=Not documented)** | **Time of follow-up (months)** |
| --- | --- | --- | --- | --- | --- | --- | --- | --- | --- | --- | --- | --- | --- |
| 1 | Mansour et al. [16] | 1985 | 5 | 1 | 1 | 2 | 2 | The eyelid mass recurred after five excisions (at the age of 12-year-old) |  | 1 | 4 | 3 | ND |
| 2 | Shields et al. [17] | 1990 | 0.25 | 1 | 2 | 1 | 1 | The eyelid mass involved the orbit | 3 | 1 | 2 | 1 | 5 |
| 3 | Chalfin and Lloyd [18] | 1998 | 24 | 2 | 1 | 1 | 1 | Eyelid mass | 2 | 1 | 1 | 3 | ND |
| 4 | Hayashi et al. [19] | 2004 | 2.58 | 1 | 2 | 1 | 2 | Eyelid mass | 4 | 1 | 1 | 1 | ND |
| 5 | Nishina et al. [20] | 2004 | 1 | 1 | 2 | 1 | 1 | The eyelid mass recurred after three excisions ((at the age of 7-year-old) |  | 1 | 1 | 1 | 24 |
| 6 | Kaur et al. [21] | 2006 | 0.83 | 1 | 1 | 2 | 1 | Eyelid mass | 9 | 1 | 2 | 1 | 8 |
| 7 | Kuruvillaet al. [22] | 2009 | 0.05 | ND | 1 | 1 | 2 | Eyelid mass | 0.6 | 1 | 2 | 2 | 3 |
| 8 | Lim et al. [23] | 2010 | 2 | 1 | 2 | 1 | 2 | Eyelid mass | 6 | 1 | 1 | 1 | 4 |
| 9 | Al-Faky [24] | 2012 | 43 | 1 | 1 | 2 | 2 | Eyelid mass |  | 0 | 1 | 3 | ND |
| 10 | Zhai and Chen [25]^#^ | 2013 | 38 | 2 | 2 | 1 | 2 | Eyelid mass | 6 | 1 | 1 | 1 | 12 |
| 11 | Chiang et al. [26] | 2014 | 46 | 2 | 1 | 1 | 2 | Eyelid mass | 12 | 1 | 1 | 3 | ND |
| 12 | Shields et al. [27] | 2014 | 0.15 | 2 | 1 | 1 | 2 | Eyelid mass | 2 | 1 | 1 | 1 | 12 |
| 13 | Samara et al. [12]^*^ | 2015 | 0.06 | 2 | 1 | 1 | 2 | Eyelid mass | 0.75 | 1 | 1 | 1 | 11 |
| 14 | Samara et al. [12]^*^ | 2015 | 0.92 | 1 | 2 | 1 | 2 | Mass involving the iris and eyelid | 11 | 1 | 5 | 3 | NA |
| 15 | Surapaneni et al. [28] | 2015 | 1 | 2 | 2 | 3 | 2 | Eyelid mass | 10 | 0 | 1 | 3 | ND |
| 16 | Rajak and Selva [29] | 2016 | 8 | 2 | 1 | 1 | 1 | Eyelid mass |  | 0 | 1 | 3 | ND |
| 17 | Chubak et al. [30] | 2019 | 0.04 | 2 | 2 | 2 | 2 | Eyelid mass |  | 0 | 2 | 1 | 11 |
| 18 | Hassan et al. [31] | 2019 | 3 | 2 | 2 | 1 | 2 | The eyelid mass did not resolve after topical steroids | 6 | 1 | 1 | 1 | 3 |
| 19 | Saifaldein et al. [32] | 2019 | 4 | 1 | 1 | 2 | 1 | Eyelid mass | 0.75 | 1 | 1 | 3 | ND |

ND, Not Documented.

^*^There were 2 cases reported in this literature.

^#^This case published in Chinese literature.
